# Effect of Glycine on Lead Mobilization, Lead-Induced Oxidative Stress, and Hepatic Toxicity in Rats

**DOI:** 10.1155/2011/430539

**Published:** 2011-07-24

**Authors:** Yolanda Alcaraz-Contreras, Lourdes Garza-Ocañas, Katya Carcaño-Díaz, Xóchitl Sofía Ramírez-Gómez

**Affiliations:** ^1^División de Ciencias Naturales y Exactas, Departamento de Farmacia, Universidad de Guanajuato, Noria Alta s/n, 36050 Guanajuato, Gto., Mexico; ^2^Departamento de Farmacología y Toxicología, Facultad de Medicina, Universidad Autónoma de Nuevo León, Gonzalitos 235 Norte, Col Mitras Centro, 64460 Monterrey Nuevo León, Mexico; ^3^Departamento de Histología, Facultad de Medicina, Universidad Autónoma de Nuevo León, Monterrey Nuevo León, Mexico; ^4^División de Ciencias de la Salud, Departamento de Medicina y Nutrición, Universidad de Guanajuato, 20 de Enero 929, 37320 León, Gto., Mexico

## Abstract

The effectiveness of glycine in treating experimental lead intoxication was examined in rats. Male Wistar rats were exposed to 3 g/L lead acetate in drinking water for 5 weeks and treated thereafter with glycine (100 and 500 mg/kg, orally) once daily for 5 days or glycine (1000 mg/kg, orally) once daily for 28 days. The effect of these treatments on parameters indicative of oxidative stress (glutathione and malondialdehyde levels), the activity of blood *δ*-aminolevulinic acid dehydratase, and lead concentration in blood, liver, kidney, brain, and bone were investigated. Liver samples were observed for histopathological changes. Glycine was found to be effective in (1) increasing glutathione levels; (2) reducing malondialdehyde levels; (3) decreasing lead levels in bone with the highest dose. However, glycine had no effect on lead mobilization when 100 and 500 mg/kg glycine were administered. In microscopic examination, glycine showed a protective effect against lead intoxication.

## 1. Introduction

Lead is a pollutant with no beneficial biological role, and its toxicity causes numerous malfunctions. The liver, kidneys, and brain are considered to be the target organs for the toxic effects [1]. Although human lead toxicity has decreased since discontinuation of the use of lead as a gasoline additive, its exposure continues to be a public health problem across the world [2]. A number of recent studies confirmed the possible involvement of reactive oxygen species in lead-induced toxicity [3, 4]. Oxidative stress has been implicated for its contribution to lead-associated tissue injury in the liver, kidneys, brain, and other organs. In this context, several studies have been conducted to determine the effect of antioxidant supplementation in lead intoxication [5–8]. Ascorbic acid, vitamin E, methionine, and *α*-lipoic acid have been administered along with lead or after lead exposure. Data suggest that antioxidants may play an important role in abating some hazards of lead, increasing lead mobilization, and providing recoveries in altered biochemical variables [9, 10].

Recently, glycine, the simplest of the amino acids, has been given a lot of importance for its antioxidant effects [11]. Shaikh and Tang [12] investigated the effect of glycine on chronic cadmium toxicity in rats and reported that glycine protects against cadmium-induced hepatotoxicity as well as nephrotoxicity by blocking the lipid peroxidation. Deters et al. [13] reported that glycine prevented damage induced in isolated perfused rat livers by some hepatotoxic agents. This protection was evaluated by the reduction of lipid peroxidation (LPO) and the increase of glutathione (GSH) levels.

Al-Neamy et al. [14] determined the effect of blood lead on the plasma levels of amino acids in exposed lead workers. They reported that nonessential amino acids such as glycine showed significantly higher values in exposed workers than in nonexposed workers. On the other hand, Sanguinetti et al. [15] reported an abnormal excretion of glycine in occupational exposure to lead workers. More recently, Okoko and Awhin [16] reported that glycine can reduce cadmium-induced alterations in culture cells. These findings support the important antioxidant role of this amino acid.

Given the reported beneficial effects of glycine in providing recovery in oxidative stress, it was considered worthwhile to determine whether therapeutic benefits could be provided if this amino acid was administered after lead exposure. There is currently no basis for recommending the use of antioxidants; in particular, their optimal doses for treating lead intoxication are not yet known.

In this paper, we evaluated the effect of glycine administration using three different doses. We also evaluated the effect of duration of treatment with the highest dose. This was done to determine the possible influence of the dose of glycine on lead mobilization and its protection in lead-induced oxidative stress and to make a correlation between these parameters and histopathological liver changes.

## 2. Materials and Methods

### 2.1. Animals

All experiments were performed using adult male Wistar rats, weighing 160 ± 20 g, housed in stainless steel cages in a temperature controlled (22°C) room maintaining a 12 h light:dark cycle. The rats were allowed standard rat chow and water ad libitum throughout the experiment. Thirty animals were randomized into six groups (*n* = 5). Group I, the negative control, was given only standard rat chow and water. Group II, the positive control, received 3 g/L lead acetate in their drinking water ad libitum for 5 weeks; for 5 days afterwards, this group received plain water. Group III received 3 g/L lead acetate in their drinking water during weeks 1–5, and glycine (100 mg/kg) was given for 5 days afterwards. Group IV received 3 g/L lead acetate in their drinking water during weeks 1–5, and glycine (500 mg/kg) was given for 5 days afterwards. Group V received 3 g/L lead acetate in their drinking water during weeks 1–5, and glycine (1000 mg/kg) was given for 28 days afterwards. Group VI, exposed to lead for 5 weeks, was the positive control; rats in this group received plain water for 28 days afterwards.

The glycine solutions were freshly prepared and were administered by gavage (stomach tube) at three doses (100, 500, and 1000 mg/kg body weight). Control animals were administered water in the same way on the same schedule. One day after the last treatment, rats were anesthetized with sodium pentobarbital and blood samples were collected with lead-free needles via intracardiac puncture. The right femur and organs (liver, both kidneys, and brain) were resected and rinsed in cold saline, weighed, and used for lead analysis and biochemical assays. Rat livers were washed in situ via the portal vein with cold saline.

### 2.2. Blood *δ*-Aminolevulinic Acid Dehydratase

The activity of blood *δ*-aminolevulinic acid dehydratase (ALAD) was assayed according to the procedure of Tomokuni [17]. Briefly, 0.2 mL of heparinized blood was mixed with 1.3 mL of distilled water and incubated for 10 min at 37°C for complete hemolysis. After adding 1 mL of standard *δ*-aminolevulinic acid, the tubes were incubated for 60 min at 37°C. The reaction was stopped after 1 h by adding 1 mL of trichloroacetic acid (TCA). An equal volume of Ehrlich reagent was added to the supernatant, and absorbance at 555 nm was recorded after 5 min.

### 2.3. Hepatic and Renal GSH

To determine hepatic and renal GSH concentration, 0.25 g of tissue sample was homogenized on ice with 0.75 mL of phosphate buffer. A 5% TCA supernatant of the homogenate was poured into the mixture with the 5,5′-bis-dithionitrobenzoic acid (Ellman reagent), and the absorbance at 412 nm was measured [18]. The GSH concentration in the samples was determined using a calibration curve obtained from a GSH standard solution.

### 2.4. Hepatic and Renal LPO

Lipid peroxidation, as estimated by the formation of malondialdehyde (MDA), was determined for hepatic and renal samples using the technique of Ohkawa et al. [19]. Briefly, the tissue samples were minced and homogenized in a ratio of 1 g of wet tissue to 4 mL of phosphate buffer (pH = 7.4). Homogenates (0.1 mL) were added into 0.2 mL of 8.1% sodium dodecyl sulfate, 1.5 mL of 20% acetic acid, 1.5 mL of 0.8% thiobarbituric acid (TBA), and 0.7 mL of distilled water. Samples were heated for 60 min at 95°C. After cooling with tap water, a mixture of *n*-butanol and pyridine (15 : 1, v/v) was added, and the mixture was shaken vigorously. Following centrifugation at 4000 rpm for 10 min, the absorbance of the organic layer was measured at 532 nm. The concentration of the TBA–MDA complex in the samples was determined using a calibration curve obtained from a 1,1,3,3,-tetraethoxypropane standard solution.

### 2.5. Blood and Tissue Lead Determination

For blood, liver, kidney, brain, and bone, wet tissue weight and volume of blood were recorded. After tissue digestion with concentrated HNO_3_ using a Microwave Digestion System (Model MDS 2000), samples were brought to a constant volume and tissue lead content was determined following the procedure standardized in our laboratory according to the NOM-199-SSA1-2000 [20] using an atomic absorption spectrophotometer (PerkinElmer Zeeman 5100).

### 2.6. Protein Determination

The Lowry method [21] was used to determine the protein content of the tissue homogenates.

### 2.7. Histopathological Changes

The liver was removed, rinsed with normal saline, and cut into small pieces. Tissue pieces were fixed in solutions and paraffin embedded. Specimens were cut into 5 *μ*m thick sections and stained with hematoxylin and eosin (H&E stain), and apoptosis measured by DNA fragmentation was quantified using TUNEL assays. We used histological sections and a commercial kit (Trevigen). Histopathological changes were observed by light microscopy. The following parameters were evaluated: (1) liver architecture, (2) cell characteristics, and (3) nuclei characteristics.

### 2.8. Statistical Analysis

Data comparisons were conducted using a one-way analysis of variance followed by Tukey's post hoc test to compare results between the different treatment groups. A difference between animals exposed and not exposed to lead (with or without glycine treatment) with *P* < 0.05 was considered significant.

## 3. Results

Lead exposure caused a significant increase in its levels in blood, brain, liver, kidney, and bone samples compared with samples from controls (Table 1). Levels of lead in the blood and the soft tissues decreased significantly in the control group that was exposed to lead for 5 weeks and then received water for 28 days (Table 1). However, the levels did not decrease to the values observed in rats not exposed to lead (Table 1).

The data demonstrate that none of the treatments was effective in mobilizing lead from blood and soft tissues except for the higher dose of glycine, which mobilized lead from the femur (Table 2).

Lead intoxication caused a 70% inhibition of ALAD activity. None of the glycine treatments returned the ALAD activity to normal values.

**Figure 1:**
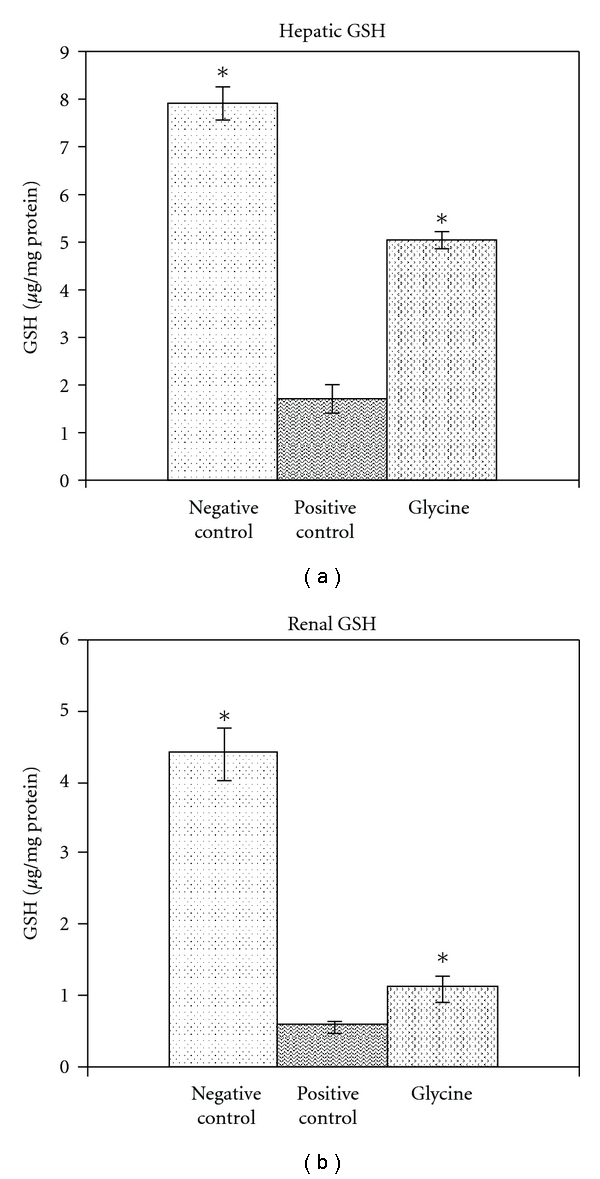
Therapeutic efficacy of glycine (100 mg/kg) on GSH levels in the liver and kidney of lead-exposed rats. Values are mean ± SE; *n* = 5. *P < 0.05 compared with positive lead control.

Lead exposure caused a decrease in levels of hepatic and renal GSH. These levels were increased with all treatments and did not show a difference between treatments in rats not exposed to lead.  Figure 1 shows GSH levels in rats treated with the lower dose of glycine.

**Figure 2:**
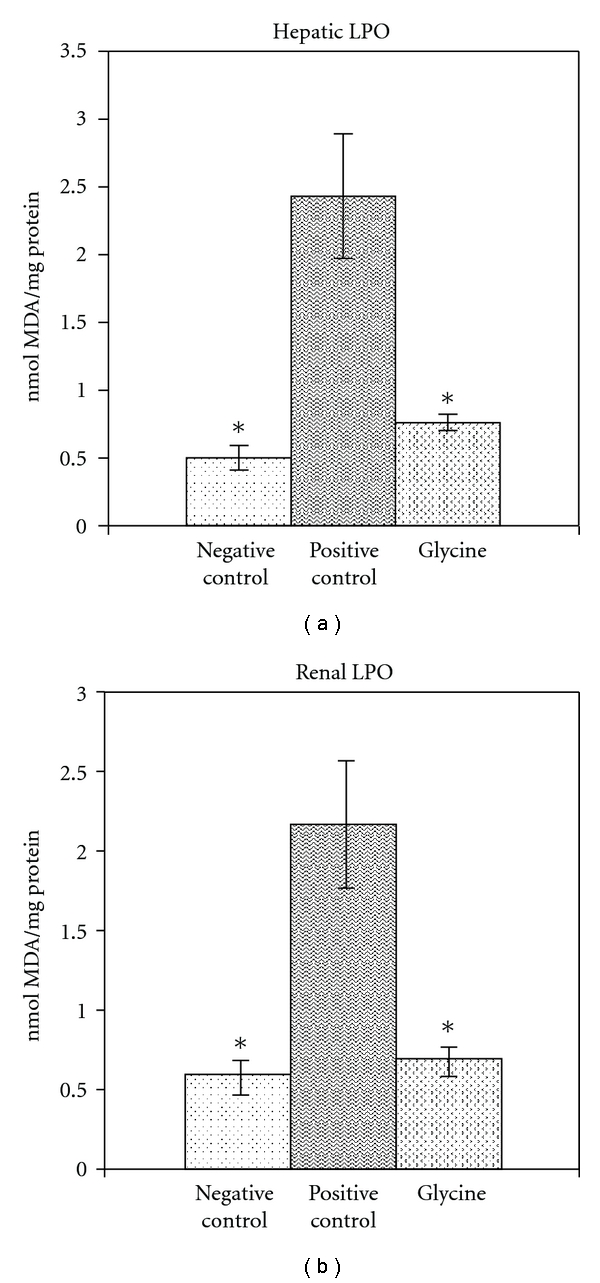
Therapeutic efficacy of glycine (100 mg/kg) on LPO levels in liver and kidney of lead-exposed rats. Values are mean ± SE; *n* = 5. *P < 0.05 compared with positive lead control.

Lead exposure caused an increase in the levels of hepatic and renal lipid peroxidation. These levels were decreased with all treatments and did not show a difference between treatments in rats not exposed to Pb.  Figure 2 shows LPO levels in rats treated with the lower dose of glycine.

**Figure 3:**
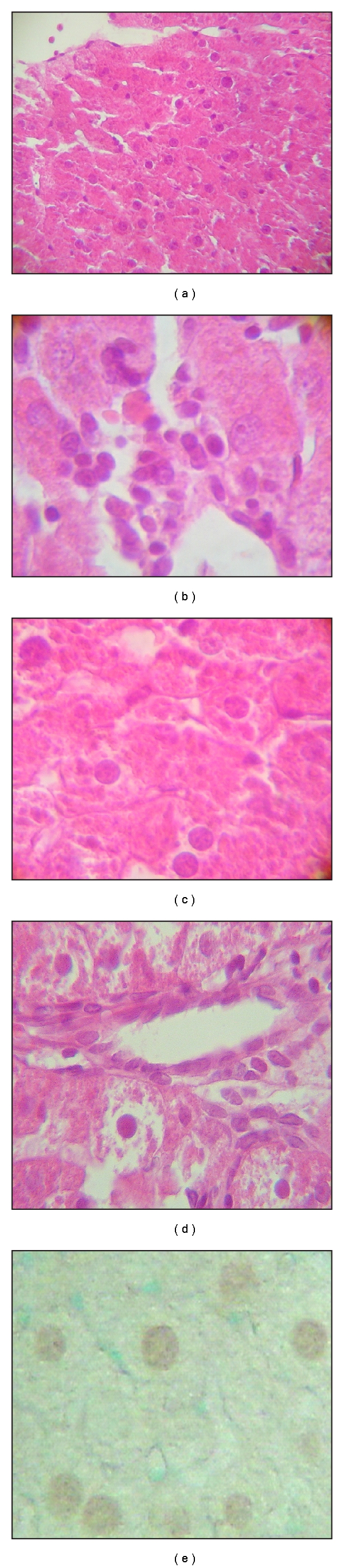
Histological lesions in the liver of rats exposed to lead. (a) Structure of a classic lobule with severe damage (H&E stain ×20). (b) Inflammatory cell infiltration (H&E stain ×64). (c) Irregular membrane cell, condensed and eosinophilic cytoplasm (H&E stain ×64). (d) Hyperchromatic and pyknotic nuclei (H&E stain ×64). (e) Apoptotic parenchyma cells (TUNEL stain ×64).

Histopathological examinations indicate that lead exposure affected the structural integrity of the liver, characterized by extensive hepatocellular degeneration or necrosis, or inflammatory cell infiltration. Congestion and dilatation of sinusoidal and portal vein branches were also observed. Hepatocytes were irregular in shape and showed changes in nuclear and cell size with an increased nuclear/cytoplasmic ratio with respect to the negative control group. The nuclei were often hyperchromatic and pyknotic. Lead also induced apoptotic fragments of DNA in liver observed through terminal deoxynucleotidyl transferase-mediated dUTP labeling (TUNEL) staining.  Figure 3 shows liver tissue from rats exposed to lead.

**Figure 4:**
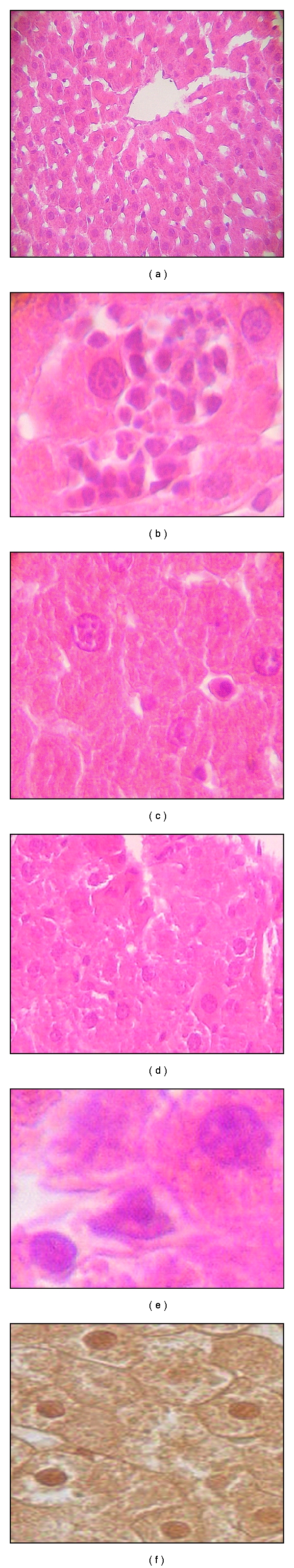
Histological lesions in the liver of rats exposed to lead and treated with glycine 100 mg/kg for 5 days. (a) Classic liver lobule, sinusoids, portal triads, and portal vein diameter were normal (H&E stain ×20). (b) Inflammatory cell infiltration (H&E stain ×64). (c) Irregular membrane cell (H&E stain ×64). (d) Necrosis (H&E stain ×64). (e) Nucleus irregular in shape (H&E stain ×64). (f) Apoptosis (TUNEL stain ×20).

Treatment of lead-exposed animals with the lowest doses of glycine showed marked improvement in histopathological changes. Sinusoidal congestion in the liver was diminished, and the portal vein diameter was normal. However, a high proportion of apoptotic nuclei were seen in parenchyma cells.  Figure 4 shows liver sections from rats exposed to lead and treated with glycine.

**Figure 5:**
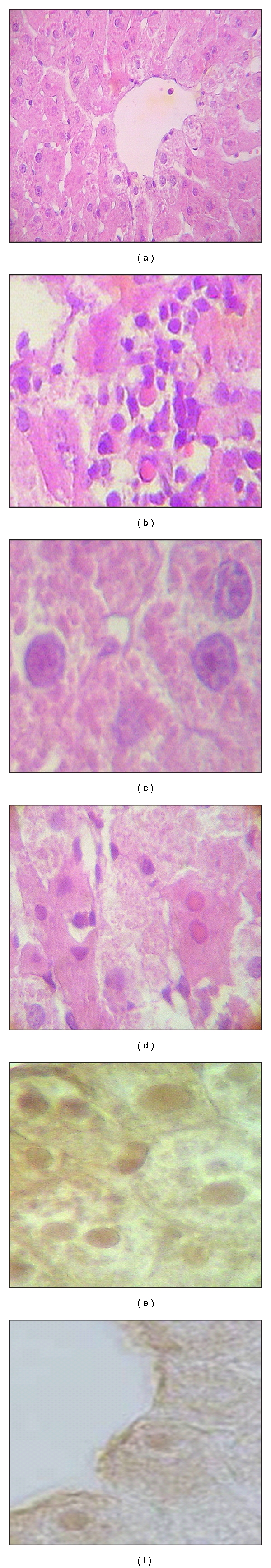
Histological lesions in the liver of rats exposed to lead and treated with glycine 1000 mg/kg for 28 days. (a) Classic liver lobule, sinusoids, portal triads, and portal vein diameter were normal (H&E stain ×20). (b) Inflammatory cell infiltration (H&E stain ×64). (c) Regular membrane cell, heterogeneous cytoplasm (H&E stain ×64). (d) Nucleus hyperchromatic with absence of basophilic staining (H&E stain ×64). (e) Apoptotic cells in the parenchyma (TUNEL stain ×64). (f) Apoptotic cells around the central vein (TUNEL stain ×64).

Treatment of lead-exposed animals with the higher doses of glycine showed marked improvement in histopathological changes. Sinusoidal congestion in the liver was diminished, and portal vein diameter was normal. Apoptotic nuclei were seen in parenchyma cells and around the central vein.  Figure 5 shows liver tissues from rats treated with glycine.

## 4. Discussion

One of the most notable observations in the present study was the significant depletion of bone lead after glycine (1000 mg/kg) administration. However, glycine administration at daily doses of 100 and 500 mg/kg for 5 days was unable to induce an effective decrease in this toxicokinetic parameter of lead. This might be because a 5-day course of treatment with glycine is inadequate to achieve optimum effects. We believe that duration of treatment as well as the glycine concentration might be important.

Although one of the major drawbacks of lead mobilization is its redistribution from bone to other critical tissues [22, 23], we did not observe an increase in the levels of lead in blood, liver, kidney, or brain tissues after glycine treatment when they were compared with their respective positive controls. This suggests that glycine can affect lead toxicokinetics in rats in a useful way.

The present study is the first reported that shows that glycine is an amino acid effective in mobilizing lead from bone. The literature has indicated that sulfur compounds such as methionine [24] increase the bioavailability of glutathione, which is useful in chelating lead, thus counteracting the toxic effects of the metal. However, glycine is not a sulfur-containing compound. It participates in GSH synthesis, and it facilitates the *γ*-glutamyl cysteinyl ethyl ester-mediated increase in intracellular GSH levels in rat hepatocytes [25]. Thus, GSH combines with certain nonnutrients, such as drugs and poisons, and promotes their biotransformation [26]. This might explain why lead bone levels are lowered as a result of the combination of GSH with lead, with subsequent excretion of the complex.

However, the mechanism by which glycine decreased the level of lead in bone could not be explained explicitly and requires further research, such as the measurement of urinary and fecal lead excretion. Experiments along these lines are already underway in our laboratory, and we are continuing the evaluation of other antioxidants in rats using different doses.

The high concentration of lead in different tissues has been associated with oxidative stress, which might be responsible, at least in part, for lead's toxic effects. We observed that lead exposure results in a marked increase in LPO in kidney and liver tissues. GSH levels were decreased in rats exposed to lead. We found that glycine therapy was effective in mitigating all of the effects of lead on parameters indicative of oxidative stress. These findings were equally observed at the three doses evaluated. On the other hand, one of the most important physiological functions of GSH is to protect the thiol groups located on some proteins and enzymes such as ALAD from oxidizing [27]. Although the three glycine treatments resulted in a significant increase in GSH levels, we did not observe reactivity of ALAD. We believe that the high levels of lead in blood in all groups treated may also explain the lack of reactivity of ALAD.

In this regard, glycine has already been shown to have protective effects against lead intoxication. Its beneficial effects could be attributed to its ability to reverse measures of oxidative stress, suggesting that glycine has an excellent antioxidant effect.

This study also showed that lead induced hepatotoxicity, as evidenced by biochemical measurements and histopathological changes that were consistent with the observations of other investigators [28]. It is of interest that despite the glycine-induced reversal of measures of oxidative stress, the histopathological examination of liver tissues confirmed that there was still some cellular damage.

In conclusion, treatment with glycine significantly decreased lead levels in bone and mitigated all of the effects of lead on parameters indicative of oxidative stress in hepatic and renal samples. These findings show that glycine has an important antioxidant activity and accelerates the elimination of lead when it is administered at a high dose. Treatment of rats with smaller and higher doses of glycine provided similar protection against lead-induced cellular liver damage and reversed completely the damage to classic liver architecture.

## Figures and Tables

**Table 1:** Lead concentration in the blood (*μ*g/dL), brain, liver, kidney, and bone (*μ*g/g) of rats. Lead was given in drinking water for 5 weeks, after which, the lead was withdrawn and the rats (1) received plain water for 5 days or (2) received plain water for 28 days.

Control groups	Blood	Brain	Liver	Kidney	Bone
Negative control	0.65 ± 0.23	0.21 ± 0.05	0.50 ± 0.17	0.20 ± 0.05	1.1 ± 0.3
Positive lead control^1^	39.2 ± 5.1^a^	1.54 ± 0.30^a^	2.3 ± 0.30^a^	10.9 ± 2.7^a^	514.5 ± 69^a^
Positive lead control^2^	24.3 ± 3.6^a,b^	0.90 ± 0.15^a,b^	1.0 ± 0.20^a,b^	6.7 ± 1.3^a,b^	407.4 ± 31.2^a,b^

^a^ *P* < 0.05 compared with negative control values.

^b^ *P* < 0.05 compared with positive lead control^1^.

**Table 2:** Lead concentration in the blood (*μ*g/dL), brain, liver, kidney, and bone (*μ*g/g) of rats. Lead was given in drinking water for 5 weeks, after which, the lead was withdrawn, and glycine (100 or 500 mg/kg) treatment was given at a daily oral dose for 5 days, or glycine (1000 mg/kg) treatment was given at a daily oral dose for 28 days.

Treatments	Blood	Brain	Liver	Kidney	Bone
Glycine (100 mg/kg)	40.5 ± 2.3	1.25 ± 0.24	2.2 ± 0.20	10.1 ± 1.3	499.9 ± 16.3
Glycine (500 mg/kg)	39.1 ± 2.1	1.41 ± 0.24	1.9 ± 0.30	9.1 ± 1.4	446.9 ± 42.8
Glycine (1000 mg/kg)	22.5 ± 1.9	0.91 ± 0.18	0.80 ± 0.40	5.9 ± 1.7	306.9 ± 35.5^a^

^a^ *P* < 0.05 compared with its positive lead control.
